# Characterization of active miniature inverted-repeat transposable elements in the peanut genome

**DOI:** 10.1007/s00122-012-1798-6

**Published:** 2012-02-01

**Authors:** Kenta Shirasawa, Hideki Hirakawa, Satoshi Tabata, Makoto Hasegawa, Hiroyuki Kiyoshima, Sigeru Suzuki, Sigemi Sasamoto, Akiko Watanabe, Tsunakazu Fujishiro, Sachiko Isobe

**Affiliations:** 1 Department of Plant Genome Research, Kazusa DNA Research Institute, 2-6-7 Kazusa-Kamatari, Kisarazu, Chiba 292-0818 Japan; 2 Peanut Plant Breeding Field, Chiba Prefectural Agriculture and Forestry Research Center, 199 He, Yachimata, Chiba 289-1113 Japan

## Abstract

Miniature inverted-repeat transposable elements (MITEs), some of which are known as active non-autonomous DNA transposons, are found in the genomes of plants and animals. In peanut (*Arachis hypogaea*), *AhMITE1* has been identified in a gene for fatty-acid desaturase, and possessed excision activity. However, the *AhMITE1* distribution and frequency of excision have not been determined for the peanut genome. In order to characterize *AhMITE1*s, their genomic diversity and transposition ability was investigated. Southern blot analysis indicated high *AhMITE1* copy number in the genomes of *A. hypogaea*, *A. magna* and *A. monticola*, but not in *A. duranensis*. A total of 504 *AhMITE1*s were identified from the MITE-enriched genomic libraries of *A. hypogaea*. The representative *AhMITE1*s exhibited a mean length of 205.5 bp and a GC content of 30.1%, with AT-rich, 9 bp target site duplications and 25 bp terminal inverted repeats. PCR analyses were performed using primer pairs designed against both flanking sequences of each *AhMITE1*. These analyses detected polymorphisms at 169 out of 411 insertional loci in the four peanut lines. In subsequent analyses of 60 gamma-irradiated mutant lines, four *AhMITE1* excisions showed footprint mutations at the 109 loci tested. This study characterizes *AhMITE1*s in peanut and discusses their use as DNA markers and mutagens for the genetics, genomics and breeding of peanut and its relatives.

**Electronic supplementary material:**

The online version of this article (doi:10.1007/s00122-012-1798-6) contains supplementary material, which is available to authorized users.

## Introduction

Transposable elements were first found in maize (McClintock 1951). They have been identified in both plants and animals (Feschotte et al. 2002). There are two types of transposable element, retrotransposons and DNA transposons. Retrotransposons move within genomes via RNA intermediates, using a ‘copy-and-paste’ mechanism. In contrast, the DNA of a DNA transposon is moved by a ‘cut-and-paste’ mechanism. The DNA transposons may be categorized into two groups, autonomous and non-autonomous elements. Autonomous elements contain regions encoding transposases which are necessary for the movement of transposons. Non-autonomous elements lack these coding sequences and must be activated by transposases supplied by autonomous elements. Most DNA transposons are flanked by terminal inverted repeats (TIRs), which function as receptor sites for the transposases (Feschotte et al. 2002). Target-site duplications (TSDs) are observed outside the TIRs.

Miniature inverted-repeat transposable elements (MITEs) are non-autonomous elements of less than 600 bp in length. There are two major families of MITE, *Tourist* and *Stowaway*, as well as several minor families (Casa et al. 2000; Casacuberta et al. 1998; Charrier et al. 1999). Plant genomes usually contain between 10^3^ and 10^5^ copies (Feschotte et al. 2002). MITE mobility was demonstrated initially in rice (Jiang et al. 2003; Kikuchi et al. 2003; Nakazaki et al. 2003) and has been reported subsequently in other eukaryotes (Momose et al. 2010). It is likely that MITEs are one of the key factors accelerating eukaryotic evolution (Naito et al. 2009), since they tend to transpose into genes or their flanking regions (Feschotte et al. 2002), which may result in the disruption or promotion of gene expression.

Peanut (*Arachis hypogaea*) is an important food and oil crop. This allotetraploid species possesses an AABB genome derived from two diploids, most likely *A. duranensis* (AA) and *A. ipaënsis* (BB) (Kochert et al. 1996). On the basis of branching habit and branch length, peanuts are categorized into two subspecies: *hypogaea* and *fastigiata*; six varieties: *hypogaea*, *hirsuta*, *fastigiata*, *vulgaris*, *aequatoriana*, and *peruviana*; and four agronomic types: Virginia, Spanish, Valencia and Southeast-runner (Krapovickas and Gregory 1994, 2007). In contrast to its large phenotypic variation, the species exhibits extremely low genetic diversity, as revealed by polymorphism analyses using restriction fragment length polymorphism and simple sequence repeat (SSR) marker systems (Burow et al. 2001; Ferguson et al. 2004; He et al. 2003; Koilkonda et al. 2011; Moretzsohn et al. 2004, 2005, 2009; Proite et al. 2007).

Patel et al. (2004) reported that, following treatment with a chemical mutagen, MITE insertion caused functional disruption of the fatty-acid desaturase-encoding gene *ahFAD2B*. This MITE did not belong to the *Tourist* or *Stowaway* families but contained 9 bp TSDs and 25 bp TIRs, and was also present in multiple copies in the genome (Patel et al. 2004). These findings were similar to those of the *Bigfoot* family in *Medicago* (Charrier et al. 1999). *AhMITE1*, which exhibits sequence similarities with the MITE reported by Patel et al. (2004), has been excised subsequently from a single locus in spontaneous and induced mutants (Gowda et al. 2010, 2011). These reports suggest that *AhMITE1* transposes or is activated by ethyl methane sulfonate, gamma irradiation, adverse environmental conditions and tissue culture.

This investigation focused on the genomic diversity of peanut *AhMITE1* and its ability to transpose to provide a better understanding of the roles played by MITEs in the peanut genome and to develop tools for genetic and genomic studies. Following the collection of genomic fragments containing *AhMITE1*s, nucleotide sequence analyses of *AhMITE1* loci indicated that these elements clustered into six subfamilies. Insertional polymorphisms were detected by PCR analyses. The genomic distribution and transposition ability of *AhMITE1* elements were also investigated. The discussion includes the potential for using these elements as DNA markers and as mutagens for advanced molecular breeding programs such as marker-assisted selection.

## Materials and methods

### Plant materials

Four peanut lines, including three Virginia types (*A. hypogaea* spp. *hypogaea* var. *hypogaea* cv. ‘Nakateyutaka’, ‘YI-0311’, and ‘Satonoka’) and one Spanish type (*A. hypogaea* spp. *fastigiata* var. *fastigiata* cv. ‘Kintoki’), were used for the construction of *AhMITE1*-enriched genomic libraries and screening of *AhMITE1*-insertion polymorphisms. The related species, *A. duranensis* (AA), *A. magna* (BB) and *A. monticola* (AABB), were also used in Southern blot analyses. For determining the transposition ability of *AhMITE1*, ‘Nakateyutaka’ seeds were treated with gamma irradiation (10 Gy/h) for 20 h at the Institute of Radiation Breeding, National Institute of Agrobiological Sciences, Japan. In June 2009, M_1_ seeds were planted in a field at the Kazusa DNA Research Institute, Japan (35º19′35′′N, 139º59′22′′E). For screening transposants, a single seed was collected from each M_1_ plant and these M_2_ seeds were planted into soil-containing pots, which were then cultivated in a plant-growth chamber. Genomic DNA was extracted from leaves using a DNeasy Plant Mini Kit (Qiagen).

### Southern blot analysis

Digoxigenin-labeled *AhMITE1* probes were prepared using a PCR DIG Labeling Mix (Roche Diagnostics, Switzerland). *AhMITE1*-containing DNA fragments from the *ahFAD2B* locus were cloned into pGEM^®^-T Easy (Promega) and used as templates for the PCR amplification of probes with the oligonucleotide primer (5′-AAGGTGGATACTACMATGAAGAT-3′). Genomic DNA was digested with *Eco*RI and separated by electrophoresis in a 1.0% agarose gel. DNA fragments were transferred to a nylon membrane (Hybond N+, GE Lifescience) and hybridized with digoxigenin-labeled probes at 65°C for 16 h. Following hybridization, membranes were washed twice with 0.5× SSC, 0.1% SDS at 60°C for 20 min. Signal detection was performed with a DIG Nucleic Acid Detection Kit (Roche Diagnostics, Switzerland).

### Construction of *AhMITE1*-enriched genomic libraries and sequence analyses

Enrichment of genomic DNA fragments containing *AhMITE1* transposons was performed as described by Nunome et al. (2006) with minor modifications. Biotin-labeled probes were prepared by PCR from the plasmid DNA used in the Southern blot analysis with the oligonucleotide primers (5′-AAGGTGGATACTACMATGAAGAT-3′) labeled at the 5′ end with biotin. Genomic DNA was digested with nine restriction enzymes, i.e., *Afa*I, *Alu*I, *Hae*III, *Hpy*CH4V, *Mse*I, *Pvu*II, *Sca*I, *Ssp*I and *Xsp*I, to enhance the number of independent clones containing *AhMITE1* sequences. Digested DNA fragments were ligated to linkers (5′-GTTTAGCCTTGTAGCAGAAGC-3′ and 5′-GCTTCTGCTACAAGGCTAAACAAAA-3′ phosphorylated at the 5′ end) using the LigaFast Rapid DNA Ligation System (Promega). Probes were then hybridized to the fragments and complementary sequences were collected using Dynal Magnetic Beads (Invitrogen). Using primers for the linker sequences, recovered DNA fragments were amplified by PCR and then ligated into pGEM-T^®^ Easy. Plasmids were introduced into *Escherichia coli* ElectroTen-blue (Stratagene) by electroporation. Following the amplification of DNA inserts with the Illustra TempliPhi DNA Amplification Kit (GE Lifescience), nucleotide sequences were determined using the BigDye Terminator Kit (Applied Biosystems) and an ABI 3730*xl* DNA sequencer (Applied Biosystems).

### Computational processing and sequence analyses

Sequence data were subjected to base-calling with the PHRED program (Ewing et al. 1998; Ewing and Green 1998). Vector and linker sequences were masked with the CROSS_MATCH program using the parameters -minmatch 10 and -minscore 18 (Ewing and Green 1998). Masked and low quality bases generating Phred scores <20 were clipped using the TRIM2 program (-q 20 - × 10) (Huang et al. 2003) and sequences >1 kb were excluded. The remaining sequences were compared with the CROSS_MATCH program (-minmatch 12 -penalty -2 -minscore 20) against *AhMITE1* sequences, and masked sequences were clipped using the TRIM2 program. Following trimming of the *AhMITE1* sequences, the remaining flanking sequences were assembled with the CAP3 program using default parameters (Huang and Madan 1999). Sequences derived from the same loci were integrated into contigs, from which representative sequences were used for subsequent analyses. Similarity searches of *AhMITE1* flanking sequences were performed against the NCBI nr (non-redundant amino acid sequences) database (http://www.ncbi.nlm.nih.gov) using the BLASTX program and an *E* value cutoff of ≤1*e* ^−4^ (Altschul et al. 1997). The top hits are summarized in Table S1. For classification of the *AhMITE1*s, a multiple sequence alignment of sequences from independent loci was performed using CLUSTALW with default parameters (Thompson et al. 1994). A dendrogram of the aligned sequences was constructed with the neighbor-joining algorithm using MEGA5 software (Tamura et al. 2011). For the analysis of polymorphic insertions, PRIMER3 software was used to design primer pairs based on *AhMITE1* flanking sequences to amplify 300–600 bp DNA fragments containing *AhMITE1* loci (Rozen and Skaletsky 2000).

### PCR amplification of *AhMITE1* sites

PCR amplifications were performed using 0.5 ng peanut genomic DNA in a 5 μl reaction mix containing 1× PCR buffer (BIOLINE, UK), 3 mM MgCl_2_, 0.04 U BIOTAQ DNA polymerase (BIOLINE, UK), 0.2 mM dNTPs and 0.8 μM of each primer. The thermal cycling conditions were as follows: 1 min denaturation at 94°C; 35 cycles of 30 s denaturation at 94°C, 30 s annealing at 58°C and 1 min extension at 72°C; and a final 3 min extension at 72°C. PCR products were separated by electrophoresis in a 10% polyacrylamide gel with TBE buffer or with a micro-tip fragment analyzer (MultiNA, Shimadzu), according to the standard protocols. Gels were stained with ethidium bromide for the detection of DNA bands under UV illumination.

## Results

### Isolation and characterization of *AhMITE1*s by Southern blot analysis and sequencing analysis

**Fig. 1:**
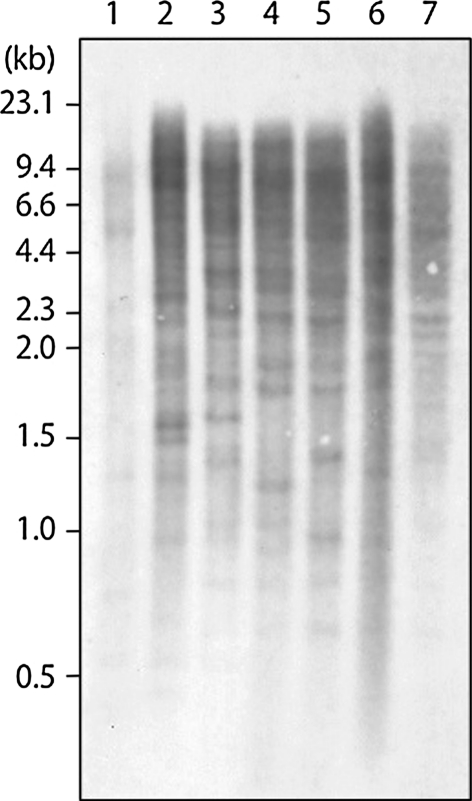
Southern blot analysis of genomic DNA from *Arachis* species using *AhMITE1* probes. 1, *A. duranensis* (AA genome); 2, *A. magna* (BB genome); 3, *A. monticola* (AABB genome); 4, *A. hypogaea* spp. *hypogaea* var. *hypogaea* cv. ‘Nakateyutaka’, Virginia type (AABB genome); 5, *A. hypogaea* spp. *hypogaea* var. *hypogaea* cv. ‘YI-0311’, Virginia type (AABB genome); 6, *A. hypogaea* spp. *hypogaea* var. *hypogaea* cv. ‘Satonoka’, Virginia type (AABB genome); 7, *A. hypogaea* spp. *fastigiata* var. *fastigiata* cv. ‘Kintoki’, Spanish type (AABB genome)

To investigate the *AhMITE1* family in the genomes of peanut and related species, Southern blot analyses were carried out using the digoxigenin-labeled *AhMITE1* fragments as probes. Multiple bands were detected in four lines of *A. hypogaea*, as well as in *A. magna* and *A. monticola*, while very faint bands were detected in *A. duranensis* (Fig. 1). The banding patterns indicated polymorphism between the four lines of *A. hypogaea* as well as between the four *Arachis* species, which suggests the presence of different *AhMITE1* insertion sites in each line.

*AhMITE1*-enriched genomic libraries were constructed using DNAs from three lines, i.e., ‘Nakateyutaka’, ‘YI-0311’, and ‘Kintoki’. Nucleotide sequences were obtained from 8,736 clones and clustering analysis of the *AhMITE1* flanking sequences indicated that 504 sequences were from independent *AhMITE1* loci (Table S1, DDBJ accession numbers: DE998420–DE998923; also see http://marker.kazusa.or.jp).

To investigate the positions of *AhMITE1* insertions in the peanut genome, the 504 flanking sequences were subjected to similarity searches against non-redundant amino acid sequences (NCBI nr: http://www.ncbi.nlm.nih.gov) using the BLASTX program. Out of the 504 sequences, 58 (11.5%) showed significant sequence similarity to reported genes (Table S1).

**Fig. 2:**
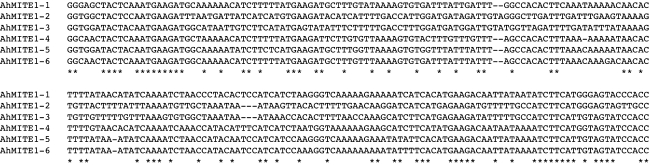
Consensus sequences of *AhMITE1* subfamilies. *Asterisks* indicate the positions of conserved bases among the six subfamilies

To predict 9 bp TSDs as reported by Patel et al. (2004), comparison analyses were performed using sequences flanking the 5′- and 3′- ends of all 504 *AhMITE1* sequences. A total of 286 pairs of TSD sequences matched completely with non-conserved AT-rich sequences (93.4%), while the remaining 218 pairs contained one to nine mismatches (Table S1). The 286 *AhMITE1*s containing completely matching TSDs were subjected to further analysis, since they presumably represented newer insertions, while the other 218 transposons contained mutations in the TSDs, TIRs and internal transposon sequences. The mean *AhMITE1* length was 205.5 bp and sizes ranged from 201 to 223 bp, while the mean GC content was 30.1%, which is lower than the *Arachis hypogaea* genome, i.e., 38.9% (DDBJ accession number: FI498696–FI503143). No protein-encoding genes were predicted among the 286 sequences, which suggests that these *AhMITE1*s are non-autonomous. Cluster analysis of the *AhMITE1* sequences (201–223 bp in length) grouped the elements into six families (AhMITE1-1 to AhMITE1-6; Fig. 2).

**Fig. 3:**
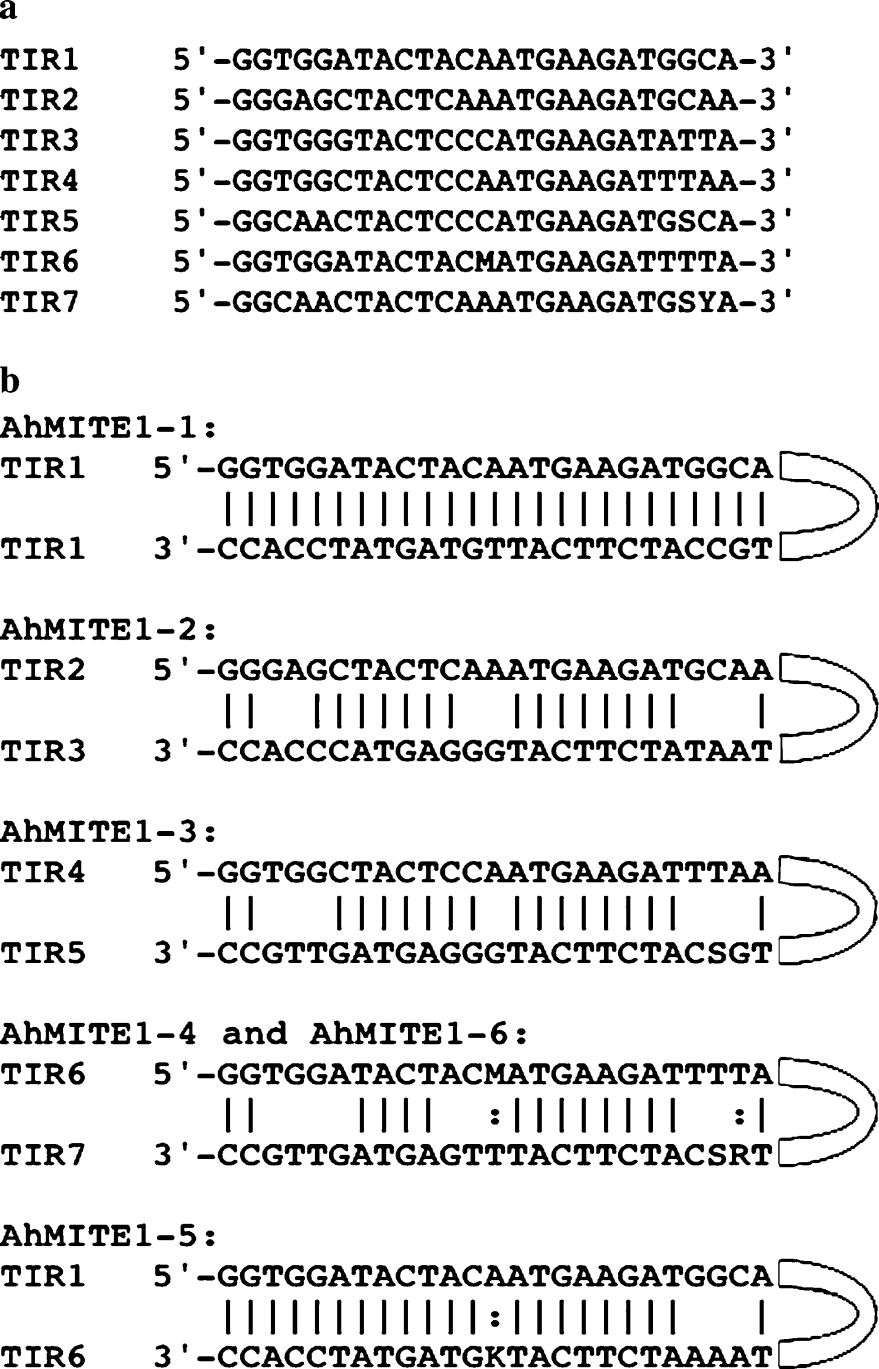
Terminal-inverted repeat sequences of *AhMITE1*s. **a** Seven TIR consensus sequences. S, M and Y show G/C, A/C and C/T, respectively. **b** Five TIR pairing patterns. Complementary matched and semi-complementary matched bases are delimited by *lines* and *colons*, respectively. Arms on the *right side* represent *AhMITE1* internal sequences. S, M, R and K show G/C, A/C, A/G and G/T, respectively

**Fig. 4:**
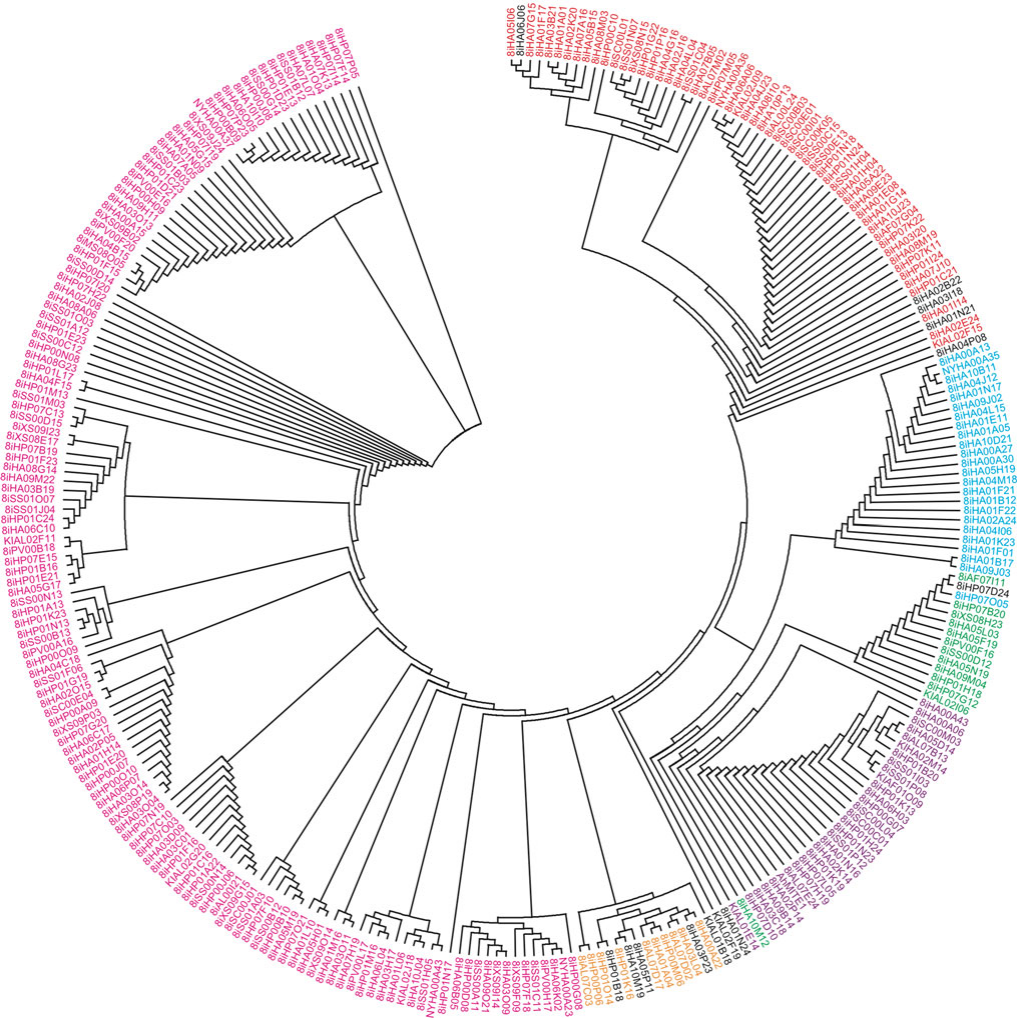
*AhMITE1* phylogenetic tree based on genetic distances calculated with the neighbor-joining method. Subfamilies of AhMITE1-1, -2, -3, -4, -5, and -6 are shown in *purple*, *pink*, *red*, *blue*, *green* and *orange letters*, respectively. *AhMITE1*s in *black letters* were not classified into any subfamilies. AhMITE1 is a sequence reported by Patel et al. (2004) (color figure online)

The ends of all 286 pairs of *AhMITE1* sequences were compared to identify TIRs, which were found to be 25 bp in length and matched the sequences reported by Patel et al. (2004). The 572 TIR sequences were classified into seven consensuses (TIR1–TIR7) and 14 unique sequences (Fig. 3a). Five combinations of seven consensus sequences were found in 273 *AhMITE1*s, with pairs of TIR1s identified in 29 sequences in addition to the original *AhMITE1* (Patel et al. 2004; Fig. 3b, Table S1). A combination of TIR2 and TIR3 was found most commonly, i.e., in 142 AhMITE1-2s, followed by TIR4–TIR5 in 55 AhMITE1-3s, TIR6–TIR7 in 24 AhMITE1-4s, TIR1–TIR6 in 13 AhMITE1-5s, and TIR6–TIR7 in 10 AhMITE1-6s (Fig. 3b; Table S1). The remaining 13 *AhMITE1* sequences exhibited unique combinations of TIRs that differed from those described above. Among these different groups, sequence diversity was greatest within the AhMITE1-2 sequences (Fig. 4).

### *AhMITE1* insertion polymorphisms among cultivated peanut lines

**Fig. 5:**
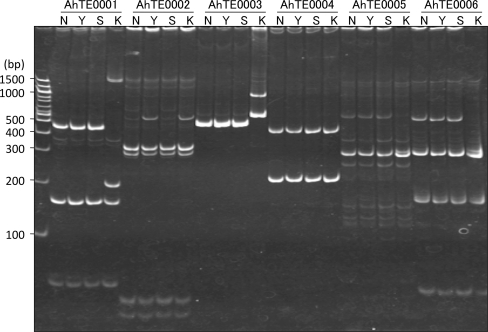
Polyacrylamide gel analysis of insertional polymorphisms in four cultivated peanut lines. Six markers were used. *N* ‘Nakateyutaka’, *Y* ‘YI-0311’, *S* ‘Satonoka’, *K* ‘Kintoki’

To investigate *AhMITE1* insertion polymorphisms among peanut lines, 504 primer pairs were designed against both flanking sequences of each *AhMITE1* (Table S1). Among the primer pairs tested, 240 and 171 generated single and double DNA bands, respectively. The double bands likely derived from homoeologous regions in the A and B genomes. Of the 411 primer pairs that produced amplicons, 169 showed polymorphism between the four lines, i.e., ‘Nakateyutaka’, ‘YI-0311’, ‘Satonoka’ and ‘Kintoki’ (Fig. 5; Table 1, Table S1). The mean number of polymorphic sites between any two lines was 90.3 (22.0%), while means of 55.0 (13.4%) and 125.6 (30.6%) were exhibited among Virginia lines and between Virginia and Spanish types, respectively (Table 1).

**Table 1:** Number and percentage of polymorphic markers among four peanut lines

Combinations	No. of polymorphic markers^a^	%
Av. among four lines	90.3	22.0
Av. among Virginia types	55.0	13.4
Nakateyutaka vs. YI-0311	58	14.1
Nakateyutaka vs. Satonoka	30	7.3
YI-0311 vs. Satonoka	77	18.7
Av. between Virginia and Spanish types	125.6	30.6
Nakateyutaka vs. Kintoki	124	30.2
YI-0311 vs. Kintoki	147	35.8
Satonoka vs. Kintoki	106	25.8

^a^The total number of primer pairs tested was 411

### Transposition ability of *AhMITE1*s in peanut

Since MITEs have been shown to activate under stress conditions (Kikuchi et al. 2003; Lin et al. 2006; Nakazaki et al. 2003; Shan et al. 2005), *AhMITE1* transposition activity was investigated in peanut mutants generated by gamma irradiation. ‘Nakateyutaka’ seeds were gamma-irradiated (200 Gy) and then planted out as an M_1_ population. Of the 206 M_1_ seeds, 125 died before or just after germination, while 14 grew but were sterile. The remaining 67 M_1_ plants, i.e., G01–G67, grew normally and produced self-pollinated seeds. A single seed was collected from each M_1_ plant and these seeds were sown as the M_2_ plant generation. Leaves from 60 M_2_ seedlings were collected for DNA analysis. Leaves from seven seedlings (G25, G33, G36, G37, G39, G40, and G41) were omitted from the analysis because the M_2_ seeds failed to germinate.

In the 60 M_2_ plants, *AhMITE1*-containing genomic regions were PCR amplified using 109 primer pairs, all of which amplified ‘Nakateyutaka’ alleles with *AhMITE1* insertions (Table S1). Four of the primer pairs amplified lower molecular weight fragments in a part of the M_2_ plants. Among the M_2_ plants, a homozygous *AhMITE1*-absent allele (G51) was identified at the AhTE0433 locus, a heterozygous mutant allele (G13) was observed at the AhTE0426 locus and two heterozygous mutant alleles (G13 and G49) were found at the AhTE0121 locus. An extraordinarily high frequency of M_2_ plants (14 of 60; G04, G06, G10, G18, G21, G23, G27, G32, G34, G45, G53, G58, G62, and G67) exhibited homozygous mutant alleles at the AhTE0047 locus. It was confirmed subsequently that some of these lines contained mutant alleles at the AhTE0047 locus prior to gamma irradiation, which indicates the presence of polymorphism within normal ‘Nakateyutaka’ lines.

**Fig. 6:**
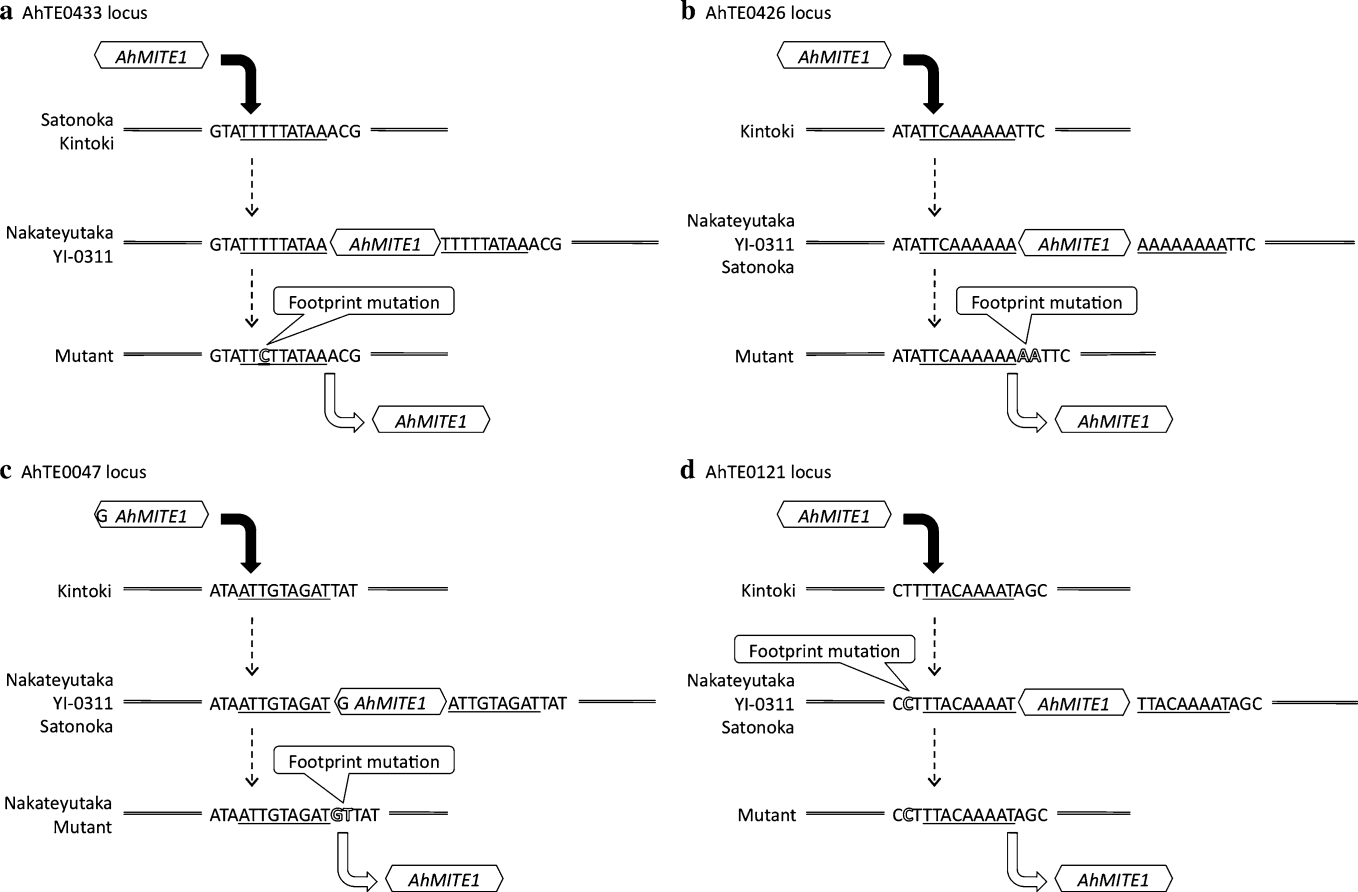
Sequences of target site duplications and flanking regions at the AhTE0433 (**a**), AhTE0426 (**b**), AhTE0047 (**c**) and AhTE0121 (**d**) loci. The nucleotides involved in target site duplications and footprint mutations are *underlined* and *outlined*, respectively. *Black and white arrows* show *AhMITE1* insertion and excision events, respectively

To examine the mutation events in the four M_2_ mutant loci, nucleotide sequence comparisons were performed between targeted regions in the 18 mutated M_2_ plants and the four peanuts lines, i.e., ‘Nakateyutaka’, ‘Kintoki’, ‘Satonoka’ and ‘YI-0311’. At the AhTE0433 locus, *AhMITE1* deletion and a single-base T/C substitution were found in the M_2_ mutant (Fig. 6a), while the AhTE0426 and AhTE0047 loci exhibited two di-nucleotide insertions and *AhMITE1* excisions (Fig. 6b, c). At the AhTE0426 locus, the AA insertion might have derived from TSD, while the GT insertion may have arisen from the end of *AhMITE1* and TSD at the AhTE0047 locus. GT insertions were observed in ‘Nakateyutaka’ lines that were not treated with gamma irradiation. At the AhTE0121 locus, a T/C mutation was identified between ‘Kintoki’ and all the other lines, while an *AhMITE1* deletion was found at the flanking sequence of the TSD in the mutant M_2_ line (Fig. 6d). This mutation may have been induced at the time of *AhMITE1* insertion. All of the investigated alleles showed *AhMITE1* excisions and base substitutions or insertions that represent probable footprint mutations.

## Discussion

In the present study, 504 *AhMITE1*s and their flanking sequences were isolated from the peanut genome and characterized. The *AhMITE1*s exhibited a mean length of 205.5 bp and a GC content of 30.1%, findings similar to those reported previously for peanut MITEs (Patel et al. 2004). Complete conservation of TIRs was observed in AhMITE1-1, but not in AhMITE1-2 to -6. The AT-rich, 9 bp TSDs were different from *Tourist* and *Stowaway* but similar to the *Bigfoot* family in *Medicago* (Charrier et al. 1999), as described by Patel et al. (2004).

Southern blot analysis revealed multiple *AhMITE1* copies in the genomes of *A. magna* (BB), *A. monticola* (AABB) and *A. hypogaea* (AABB), but not in the genome of *A. duranensis* (AA) (Fig. 1). This result suggests that *AhMITE1* elements have amplified in diploid species of the B genome and then transferred to tetraploid species. The tetraploid *A. hypogaea* was generated by hybridization between two A and B diploids. Since differences in *AhMITE1* insertion positions were observed not only among the four *Arachis* species but also between the four *A. hypogaea* lines, it is likely that *AhMITE1* transposition occurred both before and after the generation of *A. hypogaea*.

The *AhMITE1* insertion frequency into gene-containing regions was 11.5%. However, since BLASTX analysis does not detect *AhMITE1* transpositions into promoter, intron or untranscribed regions, a higher than 11.5% frequency of insertions into gene regions might be expected, which is a higher frequency than by random transposition. Due to genome size, the gene regions in the peanut genome (2.8 Gb) should be a much lower ratio than 11.5% (Yüksel and Paterson 2005) since the *Lotus japonicas* (472 Mb genome) ratio is calculated to be 9.4% (Sato et al. 2008). In maize and rice, MITEs have been reported to insert preferentially into genic regions (Bureau and Wessler 1994; Jiang et al. 2003), and similar findings have been revealed by whole genome sequencing studies in rice, sorghum and *L. japonicus* (International Rice Genome Sequencing Project 2005; Paterson et al. 2009; Sato et al. 2008). Gene function or expression may be disrupted or altered by transposition events, as well as by other genetic modifications such as genome rearrangement and duplication/deletions, and point mutations. These modifications might contribute to the divergence of many plant species, including those in the genus *Arachis*.

It is clear that at least three *AhMITE1*s, i.e., those at the AhTE0433, AhTE0426 and AhTE0121 loci, were activated following gamma irradiation. Footprint mutations were detected at all of the empty sites investigated (Fig. 6). These findings imply that *AhMITE1* transposition may be activated spontaneously or by gamma irradiation. The frequency of the de novo excision could be calculated to be 0.00023 [=3 excision events/(60 lines × 2 haploids × 109 loci)]. This value is similar to that of rice *mPing* under the normal condition (Monden et al. 2009). On the other hand, 14 lines were homozygous for empty alleles at the AhTE0047 locus, and the remaining 46 lines were homozygous for insert-containing alleles. These high frequencies of homozygous mutations suggests that *AhMITE1* excision occurred in a single or a small number of ‘Nakateyutaka’ plants during the breeding process, or when the population size was relatively small, i.e., just after this line was released in 1980 from the Chiba Prefectural Agriculture and Forestry Research Center, Japan. The later distribution of a mixed stock would explain the presence of the mutated allele within some of the ‘Nakateyutaka’ line. Although it is not possible to estimate spontaneous excision frequency at the AhTE0047 locus using the present data, this finding may indicate that *AhMITE1* remains slightly active in normal plants.

In polyploid species, reverse genetics is an effective strategy for functional genomics, as well as for mutation-based breeding. This is because single gene mutations do not necessarily confer phenotypic variation due to functional complementation by homoeologous genes. A combination or pyramiding of homoeologous mutated genes would be expected to result in phenotypic changes. In plants and animals, the target-induced local lesions in genome (TILLING) technique is a widely-used reverse genetic tool (Henikoff et al. 2004) that has been employed for allergen reduction and the improvement of quality traits in peanut (Knoll et al. 2011). In addition to TILLING, transposons and retrotransposons are also useful mutagens for reverse genetic approaches in functional genomics and mutation breedings (Gierl and Saedler 1992). In particular, MITEs tend to transpose into gene and promoter regions, which is a desirable characteristic for mutagens. The retrotransposon FIDEL is well-characterized in peanut (Nielen et al. 2010) and, like *AhMITE1*s, FIDELs may be valuable tools for functional genomics. However, at present, there is no evidence to suggest that FIDEL is active.

Although there has been considerable effort for the development of DNA markers for peanut, e.g., genomic and expressed sequence tag SSRs, the efficiency of polymorphic marker production is very low due to the narrow genetic diversity (Ferguson et al. 2004; He et al. 2003; Koilkonda et al. 2011; Moretzsohn et al. 2004, 2005, 2009; Proite et al. 2007). In both plants and animals, different patterns of MITE insertion in germplasms or individuals have also been used as DNA markers (Bonin et al. 2008; Monden et al. 2009; Grzebelus et al. 2009). Therefore, it is worth considering the use of *AhMITE1*s as DNA markers. In this study, amplified DNA fragments exhibited an approximately 200 bp difference in size, which corresponds to the size of the *AhMITE1*s (Fig. 5). This finding suggests that these polymorphisms derive from the presence or absence of *AhMITE1* at the loci, and the sequencing analysis of four loci has confirmed this prediction (Fig. 6). For the construction of linkage maps, evolutionary studies on the *Arachis* genome, and as a convenient tool for molecular breeding, the stable inheritance and the genome-wide distribution of *AhMITE1* loci are required for their use as DNA markers, while the frequency of transposition is unlikely to be a concern for linkage analysis (Monden et al. 2009).

In conclusion, this study has examined the characteristics of *AhMITE1*s in the peanut genome, as well as investigating the flanking genomic sequence, insertional polymorphisms in cultivars and transposition ability. These findings will contribute to our understanding of peanut diversity and assist in the progression of genetics, genomics and the breeding of peanut and its relatives.

## Electronic supplementary material

Below is the link to the electronic supplementary material.

Supplementary material 1 (XLS 208 kb)
